# 2215 The value of storytelling in community stakeholder feedback for clinical and translational research

**DOI:** 10.1017/cts.2018.266

**Published:** 2018-11-21

**Authors:** Laurie L. Novak, Sheba George, Kenneth Wallston, Yolanda Vaughn, Tiffany Israel, Yvonne Joosten, Neely Williams, Consuelo Wilkins, Al Richmond

**Affiliations:** 1 Vanderbilt University School of Medicine; 2 Community-Campus Partnerships for Health

## Abstract

OBJECTIVES/SPECIFIC AIMS: Community stakeholder engagement along the translational spectrum of biomedical research has been identified as a potentially crucial factor for encouraging participation among underrepresented groups, improving research relevance, and adoption of evidence into practice. Although we have developed various methods to improve communication between researchers and community stakeholders, we have not focused much attention on the manner by which community stakeholders choose to communicate with researchers in scientific feedback settings. In our PCORI funded study using Community Engagement Studios to elicit feedback on research from community stakeholders, we found that feedback from participants was frequently provided in the form of stories. This presentation aims to describe these narratives, examine their function in the feedback process and consider how a focus on these narratives enhances our understanding of community engagement for clinical and translational research. METHODS/STUDY POPULATION: The present study comes from a larger randomized, controlled methodological study. We randomized 20 investigators seeking input on their research to either a Community Engagement Studio (a panel of community members or patients) or a Translational Studio (a panel of researchers). Any faculty member or research trainee at Vanderbilt University or Meharry Medical College was eligible to participate. Each Studio panel was convened to provide project-specific input. The 153 stakeholders who participated in CE Studios were patients, caregivers, or patient advocates identified by health status, health condition, or demographic variables based on the project-based needs of the 20 researchers randomized in this project. Stakeholders include individuals with diabetes, heart failure, Parkinson’s disease, sickle cell disease, and ICU survivors. All stakeholders had experience as a partner or consultant on a research project or through serving on a research advisory board or committee. All Studios were recorded and transcribed, and experienced qualitative researchers analyzed the data. For this paper, we focus on the narrative feedback in the form of stories elicited in the CE Studios. Using qualitative methods, we coded the transcripts from the 20 CE Studios to identify stories and their functions in the feedback. Stories were defined as narratives with (a) at least one actor (b) action that unfolds over time, and (c) a realization, destination, or conflict resolution (i.e., a point of the story). For example, “I refilled my mother’s pillbox on Sunday and on Friday I found the pillbox still completely full” would be a story, however, “my mother doesn’t take her meds correctly” would not. We coded the stories for how they facilitated communication in the Studio using an open-coding style, that is we did not apply a specific theoretical framework of interaction or communication. It was possible for any given story to have more than one code applied to it; that is they were not classified in a mutually exclusive way. RESULTS/ANTICIPATED RESULTS: We found 5 major functions of stories in the Studios. Basic sender-receiver functions were noted, including responding to queries and seeking mutual understanding. The other functions served to move or add to the conversation, including adding expansion and depth, characterizing abstract concepts, and providing context, with the latter being the most frequent function of stories. Speakers provided context in a wide variety of dimensions, ranging from the context of the body to spatial and institutional contexts. These stories served to help others understand the speakers’ lived experiences. DISCUSSION/SIGNIFICANCE OF IMPACT: We often engage community members in research for their expertise with regards to their lived experiences as patients or community members, and for their experiences of healthcare and social determinants of health in particular community contexts. Yet we may expect them to share their expertise in a manner that is consistent with a scientific, explanatory framing and language. However, we know there is a difference in the way that professional researchers discuss research Versus how community members discuss research. In our PCORI study, we found that our Community Studio participants relied on storytelling as an important means to communicate their lived experiences. Their stories were often key to communicating the complex contexts of their experiences. We focus on examining these narrative practices and their functions in how community members engaged with and provided advice to researchers. This understanding may help us in: (1) Characterizing the contexts, processes, and meanings associated with community stakeholder experiences that are otherwise difficult to access. (2) Identifying community priorities relevant to research that are embedded in community narratives to better align research priorities with community needs and to improve patient outcomes. (3) Collecting insights for improving the design of community engagement activities in research. (4) Harnessing more fully the potential of community engagement in research.

